# Enhancing astaxanthin biosynthesis and pathway expansion towards glycosylated C40 carotenoids by *Corynebacterium glutamicum*

**DOI:** 10.1038/s41598-024-58700-9

**Published:** 2024-04-06

**Authors:** Vanessa L. Göttl, Florian Meyer, Ina Schmitt, Marcus Persicke, Petra Peters-Wendisch, Volker F. Wendisch, Nadja A. Henke

**Affiliations:** 1 Genetics of Prokaryotes, Faculty of Biology and CeBiTec, Bielefeld University, 33615 Bielefeld, Germany; 2 CeBiTec, Bielefeld University, 33615 Bielefeld, Germany; 3 Omics Core Facility – Proteom-Metabolom Unit (In Development), Bielefeld University, 33615 Bielefeld, Germany; 4 CZS Junior Research Group, Microsystems in Bioprocess Engineering, Institute of Process Engineering in Life Sciences, Karlsruhe Institute of Technology, 76131 Karlsruhe, Germany

**Keywords:** Astaxanthin, Glycosylated carotenoids, Pathway engineering, Fed-batch fermentation, *Corynebacterium glutamicum*, Applied microbiology, Metabolic engineering

## Abstract

Astaxanthin, a versatile C40 carotenoid prized for its applications in food, cosmetics, and health, is a bright red pigment with powerful antioxidant properties. To enhance astaxanthin production in *Corynebacterium glutamicum*, we employed rational pathway engineering strategies, focused on improving precursor availability and optimizing terminal oxy-functionalized C40 carotenoid biosynthesis. Our efforts resulted in an increased astaxanthin precursor supply with 1.5-fold higher β-carotene production with strain BETA6 (18 mg g^−1^ CDW). Further advancements in astaxanthin production were made by fine-tuning the expression of the β-carotene hydroxylase gene *crtZ* and β-carotene ketolase gene *crtW*, yielding a nearly fivefold increase in astaxanthin (strain ASTA**), with astaxanthin constituting 72% of total carotenoids. ASTA** was successfully transferred to a 2 L fed-batch fermentation with an enhanced titer of 103 mg L^−1^ astaxanthin with a volumetric productivity of 1.5 mg L^−1^ h^−1^. Based on this strain a pathway expansion was achieved towards glycosylated C40 carotenoids under heterologous expression of the glycosyltransferase gene *crtX*. To the best of our knowledge, this is the first time astaxanthin-β-d-diglucoside was produced with *C. glutamicum* achieving high titers of microbial C40 glucosides of 39 mg L^−1^. This study showcases the potential of pathway engineering to unlock novel C40 carotenoid variants for diverse industrial applications.

## Introduction

Astaxanthin (3,3′-dihydroxy-β, β-carotene-4,4′-dione) is a naturally occurring red C40 carotenoid with diverse industrial benefits found in limited quantities in nature^1^. Derived from its structure it is categorized as an oxy-functionalized carotenoid with a keto and hydroxyl group on each ionone ring^2^. Natural as well as synthetic astaxanthin is commonly used as feed and food colorant and natural astaxanthin as an antioxidant in cosmetics^3^. In recent years, potential medical applications of astaxanthin gained a lot of interest because of its anti-inflammatory, anti-apoptotic and anti-tumor properties^4,5^. Biotechnological approaches, particularly involving bacteria and yeast, have been developed to meet the high market demand for astaxanthin with an annual growth rate of 16.8%^6^.

Besides some natural astaxanthin producing microorganisms, like bacteria from the genus *Paracoccus sp*.^7^, the yeast *Xanthophyllomyces dendrorhous* , formerly known as *Phaffia* *rhodozyma*^8^ or the green algae *Haematococcus pluvialis*^9^, both pro- and eukaryotic microorganisms like *Escherichia coli*^10^, *Yarrowia lipolytica*^11^*,* or *Corynebacterium glutamicum*^12^, have been genetically modified to produce astaxanthin efficiently for industrial production purposes.

**Figure 1:**
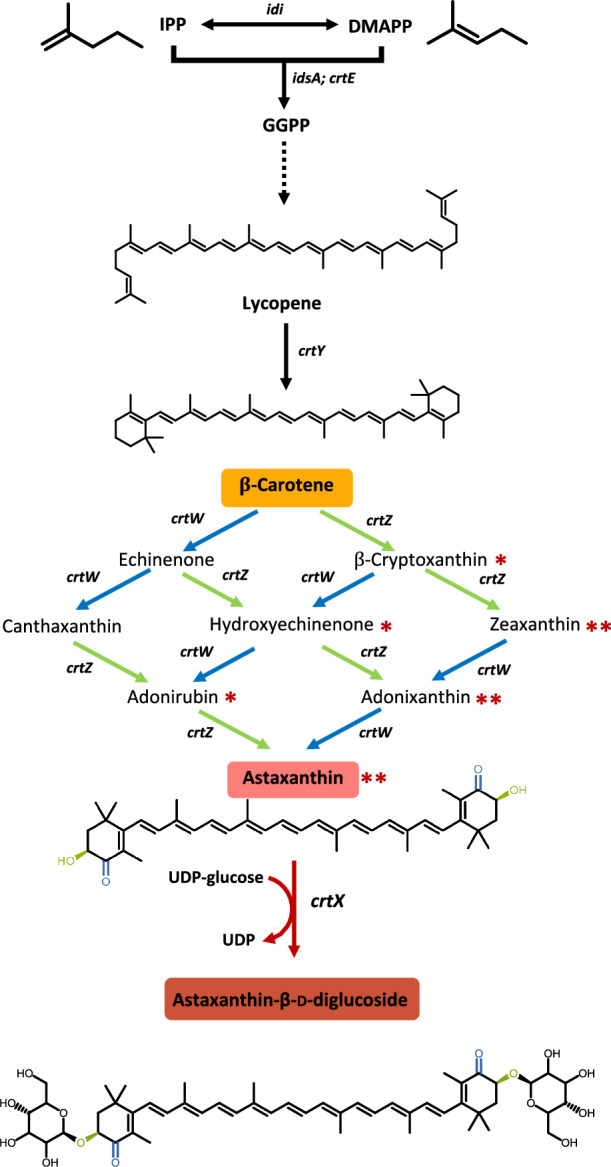
Scheme for astaxanthin and astaxanthin-β-d-diglucoside biosynthesis. Genes encoding for the enzymes are depicted next to the reaction. *idi*: IPP isomerase, *idsA*: geranylgeranyl pyrophosphate synthase, *crtE*: geranylgeranyl pyrophosphate synthase, *crtY*: lycopene β-cyclase, *crtZ*: β-carotene hydroxylase, *crtW*: β-carotene ketolase and *crtX*: glycosyltransferase. The chemical structures of IPP, DMAPP, lycopene, β-carotene, astaxanthin and astaxanthin-β-d-diglucoside are shown. Depicted in blue are the reactions from β-carotene ketolase and the introduced keto groups in astaxanthin. Depicted in green are the reactions from β-carotene hydroxylase and the introduced hydroxy groups in astaxanthin. Glycosyltransferase catalyzes the enzymatic reaction that adds specific sugar molecules to the hydroxy groups of carotenoids, here depicted for glucose added to both hydroxy groups of astaxanthin resulting in astaxanthin-β-d-diglucoside. Carotenoids with one hydroxy group are highlighted with one red star, whereas carotenoids with two hydroxy groups are highlighted with two red stars. IPP: isopentenyl pyrophosphate, DMPP: dimethylallyl pyrophosphate, GGPP: geranylgeranyl pyrophosphate.

Carotenoids are derived from isopentenyl pyrophosphate (IPP) and its isomer dimethylallyl pyrophosphate (DMAPP)^13^. The C5 compounds IPP and DMAPP are the precursors of subsequent chain elongation reactions leading to the C20 carotenoid precursor geranylgeranyl pyrophosphate (GGPP)^14^. Most carotenoids are based on a C40 backbone, which is formed by the condensation of two molecules of GGPP, resulting in lycopene^15^. Lycopene can be converted to the orange pigment β-carotene by lycopene β-cyclase^16^. Astaxanthin production is then enabled by the expression of the β-carotene hydroxylase gene *crtZ* and the β-carotene ketolase gene *crtW*^17^ (Fig. 1). Pathway engineering strategies by overexpressing of the β-carotene ketolase or hydroxylase genes^18^, balancing their expression^19^, translational fusions or modularized complexes of carotenogenesis genes^20–22^ as well as blocking of competing pathways have been shown to be effective for increasing astaxanthin production^23^. Moreover also bioprocess parameters such as temperature, pH^24^, light^25^ and process optimization have been used to enhance astaxanthin production^26,27^.

In nature, numerous hydrophobic compounds such as lipids and terpenes undergo glycosylation catalyzed by glycosyltransferases, resulting in the generation of products that exhibit improved water solubility^28^. Glycosylated carotenoids are a class of modified pigments commonly found in nature, characterized by the addition of sugar molecules to their structure. This modification plays a crucial role in enhancing the stability, solubility, and bioavailability of carotenoids, which extends their range of potential applications in industries^29,30^. For glycosylated astaxanthin it was demonstrated to exhibit improved solubility and bioavailability compared to its unmodified counterpart^31^. Glycosylated zeaxanthin showed a 3.5-fold increased singlet oxygen quenching (KQ) value than zeaxanthin^32^. In nature, glycosylated carotenoids such as zeaxanthin-glucoside^33^, astaxanthin-glucoside^34^, adonixanthin-glucoside^35^ and the C50 decaprenoxanthin-diglucoside^36^ are prevalent in various organisms, including plants, algae, and some bacteria. Glycosyltransferases, encoded by the *crtX* gene, constitute a diverse enzyme family that can transfer sugar molecules to carotenoid hydroxyl groups. The enzyme utilizes UDP-glucose as the sugar donor. The process of astaxanthin glycosylation involves transferring a glucose molecule from UDP-glucose to the hydroxyl group of the ß-ionone ring of astaxanthin, resulting in astaxanthin-β-D-diglucoside^35^ (Fig. 1). Notably, glycosylated astaxanthin has been successfully produced in *E. coli*^37^ and the yeast *Yarrowia lipolytica*^20^.

*Corynebacterium glutamicum*, a non-pathogenic Gram-positive soil bacterium, has emerged as a promising microorganism for industrial applications^38^. Initially recognized for its ability to produce the amino acid glutamic acid^39^, which found widespread use in the food and pharmaceutical industries, *C. glutamicum* has been engineered to expand its repertoire of valuable compounds^40–42^. Its rapid growth rate, capacity to utilize diverse carbon sources, metabolic versatility, established genetics, and safety for industrial use make it an attractive candidate for biotechnological applications^38,43^. *C. glutamicum* is a natural producer of the rare yellow C50 carotenoid decaprenoxanthin and its glucosides^36^. It has been metabolically engineered for efficient production of various non-natural C40 carotenoids including astaxanthin^12,44^. Recently, a screening of structurally distinct lycopene β-cyclases revealed that higher astaxanthin production can be achieved with cytosolic lycopene β-cyclase from *Synechococcus elongatus* and membrane-bound heterodimeric lycopene β-cyclase from *Brevibacterium linens*^45^. However, upon expression of β-carotene hydroxylase or β-carotene ketolase genes, a reduction of total carotenoid occurs, which might be due to a yet to be defined feedback-inhibition of the carotenoid pathway upstream of β-carotene^45^.

In this study, rational pathway engineering strategies were applied to increase astaxanthin production specifically with *C. glutamicum*. Furthermore, the best astaxanthin producing strain was transferred to a 2 L fed-batch fermentation to show robustness in a stirred bioreactor system. The here presented optimized astaxanthin producing strain proved to be suitable for a pathway extension towards the production of glycosylated C40 carotenoids with heterologous glycosyltransferase CrtX.

## Results

### Precursor pathway engineering for improving astaxanthin biosynthesis in *C. glutamicum*

**Figure 2:**
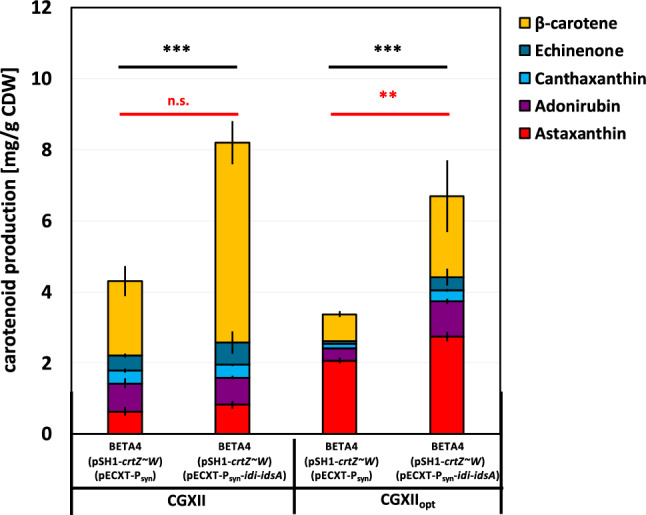
Increasing total carotenoid production by expression of *idi* and *idsA* and adaption of media for increased astaxanthin conversion. Production profiles of engineered *C. glutamicum* strains with β-carotene producing strain BETA4 expressing plasmid (pSH1-*crtZ* ~ *W*) and empty vector (pECXT-P_syn_) as a control for established astaxanthin production in *C. glutamicum*. Production yields mean values and standard deviations of three triplicate cultivations are given. Cells were grown in 40 g L^−1^ glucose CGXII minimal medium or CGXII_opt_ for 48 h in a volume of 10 mL in shaking flasks. For the total carotenoid production (black stars) and the astaxanthin production (red stars) significance was calculated with a students’ t-test p < 0.001 (***), between the strains overexpressing *idi* and *idsA* and the control strain BETA4 (pSH1-*crtZ* ~ *W*) (pECXT-P_syn_).

The astaxanthin producing *C. glutamicum* strain ASTA* was used as a starting point of this study^22^. This strain was constructed on the basis of the pro-phage cured strain MB001^46^ by blocking the pathway towards decaprenoxanthin (Δ*crtEb/*Δ*crtY*_*ef*_) resulting in a lycopene producing strain^47^. The overproduction of carotenogenesis genes *crtEBI*, together with increased supply of the precursors DMAPP and IPP increased lycopene production^47^. The production was further optimized by the deletion of the gene coding for the transcriptional regulator CrtR^48^. CrtR (cg0725) negatively regulates the *crt* operon and therefore controls carotenoid synthesis in *C. glutamicum*. Production of precursor β-carotene was achieved by genomic integration of lycopene β-cyclase from *P.* *ananatis*^12^. By expressing β-carotene hydroxylase and β-carotene ketolase from *F. pelagi*, astaxanthin production was first established in *C. glutamicum*^12^, which could be enhanced by the translational fusion protein CrtZ ~ W^22^. This strain BETA4 (pSH1-*crtZ* ~ *W*), named ASTA*, has a 10 amino acid (aa) flexible linker (GGGGSGGPGS) and produced 0.7 mg (g CDW)^−1^ (10 mg L^−1^) of astaxanthin with a total carotenoid production of 3.6 mg (g CDW)^−1^ (Fig. 2). This being 22% of astaxanthin of total carotenoid production, which shows inefficient conversion from precursor β-carotene to astaxanthin^22,49^. This strain was used as the parent strain for further improvement.

To increase astaxanthin production in *C. glutamicum* different strategies can be applied, like increasing precursor supply or improving conversion from precursors to final product. As IdsA was shown to be the major geranylgeranyl diphosphate synthase in *C. glutamicum*^50^ synthesizing the reaction from IPP and DMAPP to GGPP, the C20 building block of C40 carotenoids, *idsA* was chosen for overexpression together with *idi*, encoding for the isopentenyldiphosphate isomerase, required for isomerization of IPP and DMAPP, to increase the precursor supply. The strategy for overexpression of *idi* and *idsA* was demonstrated before^12^, but here we tested an optimized expression system, with *idi* and *idsA* expression as a synthetic operon on plasmid pECXT-P_syn_^51^ under the constitutive promotor P_syn_. As a control production strain BETA4 (pSH1-*crtZ* ~ *W*) with additional expression of the empty vector pECXT-P_syn_^51^ was cultivated. Strain BETA4 (pSH1-*crtZ* ~ *W*) (pECXT-P_syn_-*idi-idsA*) produced comparable amounts of astaxanthin as the control strain with 0.8 mg (g CDW)^−1^ (vs. 0.7 mg (g CDW)^−1^), but total carotenoid production was significantly increased twofold reaching 8.3 mg (g CDW)^−1^. This increase was mainly because of significant increase of β-carotene compared to the control strain (Fig. 2). The increase in total carotenoids results in a decreased astaxanthin ratio of 9%.

Meyer et al.^52^ could show that adaptation of media by optimizing the trace salt concentrations with less manganese (0.25 x) and more iron (2.5 x) compared to normal CGXII trace salts, provides better conversion from precursors towards astaxanthin, therefore we have chosen media CGXII_opt_ for astaxanthin production. This media increased astaxanthin production in strain BETA4 (pSH1-*crtZ* ~ *W*) (pECXT-P_syn_) to 2.0 ± 0.1 mg (g CDW)^−1^, which was further increased by overexpression of *idi* and *idsA* to 2.7 ± 0.1 mg (g CDW)^−1^, as total carotenoids were also increased in this strain to 7.0 mg (g CDW)^−1^ (Fig. 2).

### Optimization of the fusion enzyme CrtZ ~ W for astaxanthin biosynthesis in *C. glutamicum*

**Figure 3:**
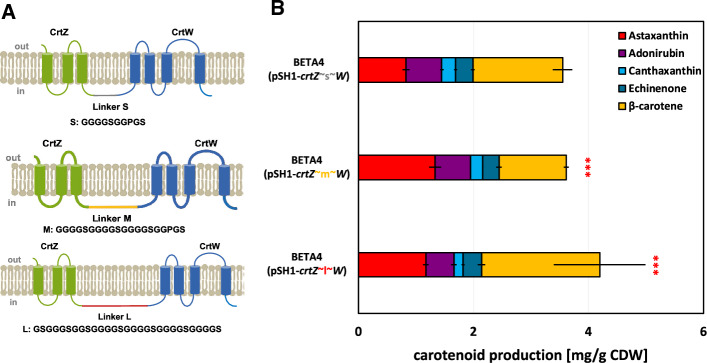
Testing different linker sizes between β-carotene hydroxylase CrtZ and β-carotene ketolase CrtW in the translational fusion protein. (**A**): scheme of constructed fusion proteins CrtZ ~ W. Transmembrane helices were predicted with DeepTMHMM^54^. CrtZ comprises 3 TMH with an extracellular N-terminus and CrtW comprises 4 TMH with both termini being intracellular. Sequence of linker sizes small (S), middle (M) and large (L) is given. (**B**): production profiles of engineered *C. glutamicum* strains with different linker sizes between CrtZ and CrtW for astaxanthin production. Production yields mean values and standard deviations of three triplicate cultivations are given. Cells were grown in 40 g L^−1^ glucose CGXII minimal medium for 48 h in a volume of 10 mL in shaking flasks. For the astaxanthin production significance was calculated with a students’ t-test *p* < 0.001 (***), in comparison to the strain with small linker size.

Different linker sizes can alter the distance between the essential proteins CrtZ and CrtW and larger linkers were tested in other organisms to increase astaxanthin production together with reducing accumulation of intermediates^53^. As the fusion of CrtW ~ Z did not lead to astaxanthin production in *C. glutamicum*^22^, we only tested CrtZ ~ W with different linker sizes. A 20 aa sized linker (middle sized linker CrtZ ~ m ~ W; GGGGSGGGGSGGGGSGGPGS) and a 29 aa sized longer linker (CrtZ ~ l ~ W) were tested for improved astaxanthin production (Fig. 3A).

Astaxanthin production was significantly increased by 60% through the usage of the middle-sized 20 aa flexible linker to 1.3 mg (g CDW)^−1^ (Fig. 3B). CrtZ ~ m ~ W increased conversion from β-carotene towards astaxanthin, as this was the only carotenoid to be reduced in comparison to the control. CrtZ ~ l ~ W with the larger 29 aa linker also increased astaxanthin by 30% towards 1.1 mg (g CDW)^−1^ (Fig. 3B), but did not increase astaxanthin further in comparison to the middle-sized linker. Thus, CrtZ ~ m ~ W was chosen in the following experiments for astaxanthin production.

After identification of two strategies to improve either astaxanthin conversion or precursor supply we chose to engineer the β-carotene producer strain BETA4^48^ to achieve a genetically stable strain that produces more precursor β-carotene. For reducing additional plasmid burden to the strain, we opted for genomic exchange of the *idi* promotor^55^ to the synthetic promotor P_syn_ with integration of additional *idsA* in the *idi* locus in β-carotene producing BETA4 strain. This resulted in strain BETA4Δ*idi*::P_syn_-*idsA-idi* (named BETA6)*,* which has two copies of *idsA* in the genome with stronger promotor P_syn_ for the *idi* locus. The β-carotene production of this strain was compared to parent strain BETA4^12^ and BETA4 (pECXT-P_syn_-*idi-idsA*). The β-carotene production was significantly increased with plasmid-based expression of *idi-idsA* to 19.8 ± 2.0 mg (g CDW)^−1^ (+ 36%). BETA6 produced comparable amounts to the plasmid-based overexpression with 18.0 ± 2.0 mg (g CDW)^−1^, which is 28% than BETA4 (Table S1). This results in an increased potential for astaxanthin production of *C. glutamicum* with BETA6.

### Balancing of terminal *crtZ*, *crtW* and *crtZW* expression results in biosynthesis of astaxanthin as major carotenoid

**Figure 4:**
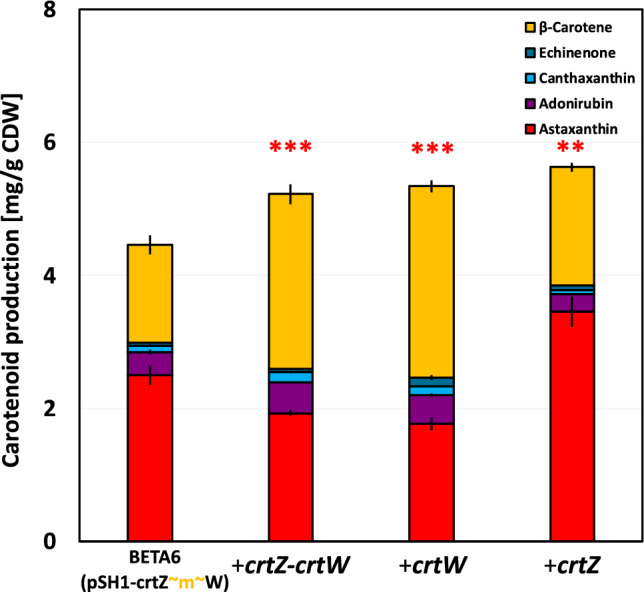
Astaxanthin production of *C. glutamicum* strains with additional expression of β-carotene hydroxylase and ketolase genes. (**A**) carotenoid contents in mg (g CDW)^−1^ (**B**) carotenoid titer in mg L^−1^. Production yields mean values and standard deviations of three triplicate cultivations are given. Cells were grown in 40 g L^−1^ glucose CGXII_opt_ minimal medium for 48 h in a volume of 10 mL in shaking flasks for astaxanthin production. For astaxanthin production (red stars) significance was calculated with a students’ t-test *p* < 0.01 (**), *p* < 0.001 (***), in comparison to the control strain BETA6 (pSH1-*crtZ* ~ *m* ~ *W*).

The two identified pathway engineering strategies, namely an improved precursor supply and an optimized terminal fusion protein, were combined in strain BETA6 (pSH1-crtZ ~ m ~ W) (Fig. 4). The new engineered strain improved astaxanthin production to 2.5 ± 0.1 mg (g CDW)^−1^ astaxanthin (31 mg L^−1^) cultivated in CGXII_opt_ (Fig. 4). As many precursors of the astaxanthin biosynthesis pathway are accumulating, a strategy for a terminal pull towards astaxanthin as the target product was aimed at by a balancing of terminal genes *crtZ* and *crtW*. The terminal pathway balancing was conducted by additional constitutive expression of *crtZ* and/or *crtW* as single genes or as a synthetic operon from the plasmid pECXT-P_syn_^51^. Interestingly the astaxanthin production dropped to 1.9 ± 0.1 mg (g CDW)^−1^ (11 mg L^−1^) in the case of an additional expression of *crtZ-crtW* (Fig. 4, Table 1). This astaxanthin content is comparable to the initial strain BETA4 (pSH1-*crtZ* ~ *s* ~ *W*) with 10 mg L^−1^ (Fig. 3) at the beginning of this study. The additional expression of *crtW* showed a significant reduction of astaxanthin to 1.8 ± 0.1 mg (g CDW)^−1^ (18 mg L^−1^). However, both strains resulted in significant higher β-carotene accumulation of 2.6 or 2.8 mg (g CDW)^−1^, respectively (Fig. 4, Table 1).

**Table 1:** Overview of astaxanthin production with BETA6 (pSH1-crtZ ~ m ~ W) derivatives with pECXT99A overexpressing *crtZ-crtW/crtW/crtZ* under fed-batch fermentation conditions.

	Titer [mg L^**−**1^]	Vol. productivity [mg L^**−**1^ h^**−**1^]	Yield [mg g^**−**1^ glucose]	Content [mg (g CDW)^**−**1^]
Batch process in shaked flasks				
+ *crtZ-crtW*	17	0.35	0.42	1.9
+ *crtW*	11	0.23	0.28	1.8
+ *crtZ*	39	0.81	0.98	3.5
Fed-batch process in stirred bioreactor				
+ *crtZ-crtW*	48	0.7	0.16	1.0
+ *crtW*	50	0.7	0.16	1.3
+ *crtZ*	103	1.5	0.34	1.8

Maximal titer, volumetric productivity, product yield and astaxanthin content are given from batch process in shaked flasks or fed-batch process in stirred bioreactors.

On the other hand, the additional expression of *crtZ* in strain BETA6 (pSH1-*crtZ* ~ *m* ~ *W*) (pECXT-P_syn_-*crtZ*) improved astaxanthin production significantly to 3.5 ± 0.1 mg (g CDW)^−1^ (39 mg L^−1^). In this strain the precursors adonirubin, canthaxanthin and echinenone were efficiently converted to astaxanthin, whereas β-carotene content was comparable to the control strain (Fig. 4, Table 1). The strain was named ASTA**, as astaxanthin was the dominant carotenoid with 72% with an improved astaxanthin production of 5.8-fold in comparison to the initial strain ASTA*.

### Transfer of astaxanthin production in 2 L fed-batch bioprocess

**Figure 5:**
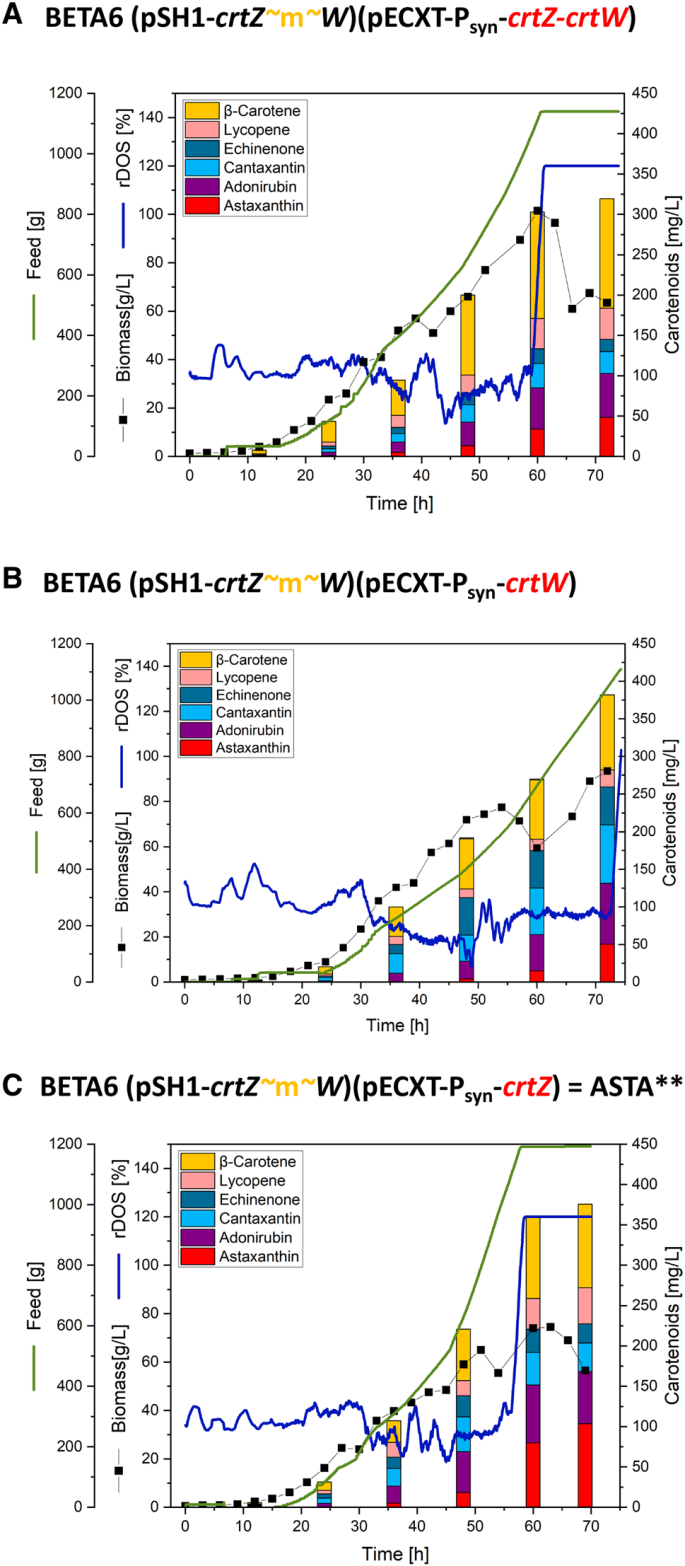
Fed-Batch fermentations of astaxanthin producer strains. Fed-batch fermentation with *C. glutamicum* astaxanthin producer strains grown in 1 L HCDC as batch medium, fed with 1 L 600 g L^−1^ glucose at pH 8 with an initial aeration rate of 0.5 vvm. Given for all fermentations are the following fermentation parameters over time: Carotenoid profile, biomass (black squares), feed intake (green line), moving average rDOS over 2 h (dark blue line). (**A**) Fermentation process of strain BETA6 (pSH1-*crtZ* ~ m~ *W*) (pECXT-Psyn-*crtZ-crtW*). (**B**) Fermentation process of strain BETA6 (pSH1-*crtZ* ~m~ *W*) (pECXT-Psyn-*crtW*). (C): Fermentation process of strain BETA6 (pSH1-*crtZ* ~m~ *W*) (pECXT-Psyn-*crtZ*) = ASTA**.

For industrial application, stable productions at larger scale are necessary. To test the strain robustness in lab-scale stirred bioreactor, the astaxanthin producing *C.* *glutamicum* strains from Fig. 4 were transferred to 2 L fed-batch fermentation (Fig. 5), as performed by Meyer et al.^27^. As a transfer from a shaken cultivation system (e.g. flask) to a stirred bioreactor system is fundamental, we opted for testing all three strains expressing additionally both CrtZ-CrtW, only CrtW or only CrtZ in a fed-batch process. All fermentations were run for around 70 h until the complete feed of glucose was consumed. Production parameters for astaxanthin are listed in Table 1.

The fermentation titers show the same trends in comparison to the shake flask results of the chapter before, where strain ASTA** was superior for astaxanthin production in comparison to the strain with additional expression of *crtW* or *crtZ-crtW*. Both of the latter strains produced comparable titers of astaxanthin with 50 mg L^−1^ or 48 mg L^−1^, respectively (Fig. 5, Table 1). This resulted in a volumetric productivity of 0.7 mg L^−1^ h^−1^ and a yield of 0.16 mg g^−1^ glucose each. However, for strain ASTA** a significant higher titer of astaxanthin with 103 mg L^−1^ (Fig. 5) was achieved that is approximately twofold higher than in the previous cultivation system of shaking flasks (Table 1), but the cellular astaxanthin content was reduced to 1.8 mg (g CDW)^−1^. The fermentation had a volumetric productivity of 1.5 mg L^−1^ h^−1^ and a yield of 0.34 mg g^−1^ glucose (Fig. 5, Table 1).

Considering the total carotenoid titer during the fed-batch processes, it can be stated that high total carotenoid titers (given in astaxanthin equivalents) of 340 to 420 mg L^−1^ were achieved with all three strains (Fig. 5), showing that besides the direct precursor adonirubin also the other precursors like canthaxanthin, echinenone, lycopene and β-carotene were accumulated with astaxanthin of up to 26% of total carotenoids (Fig. 5).

### Pathway expansion towards glycosylated C40 carotenoid biosynthesis in *C. glutamicum*

Based on the enhanced astaxanthin production (strain ASTA**), the aim was to extend the biosynthesis pathway to glycosylated carotenoids. As it was shown that balancing of the expression of terminal genes in the astaxanthin biosynthesis was beneficial for a metabolic pull, consequently it could be hypothesized that the production of glycosylated carotenoids would pull the carbon flux even more to this pathway and could lead to higher conversion of precursor towards astaxanthin and its glucosides. As key biosynthetic enzymes are often inhibited by their end products, glycosylation of astaxanthin could bypass the feedback inhibition of free astaxanthin on its pathway and increase total carotenoid production, as has been found for esterified astaxanthin^56^.

To produce glycosylated carotenoids the gene *crtX* encoding a glycosyltransferase had to be expressed. Two genes from two different organisms were chosen to test, firstly *crtX* from *P. ananatis*^33^ (uniprot: P21686) and *crtX* from *F.* *pelagi* (uniprot: A0A0P0Z8H5), both being annotated as zeaxanthin glycosyltransferases. We chose *crtX* from *P.* *ananatis* as an established gene for production of glycosylated C40 carotenoid^20,37^ and *crtX* from *F.* *pelagi*, as β-carotene hydroxylase and β-carotene ketolase from *F.* *pelagi* for heterologous astaxanthin production in *C. glutamicum* produced highest amounts of astaxanthin. The genes were expressed on an IPTG inducible plasmid in strain ASTA** (Table S3). The strain expressing *crtX* from *P.* *ananatis* was named ASTAGLYC_Pa_ and the strain expressing *crtX* from *F.* *pelagi* was named ASTAGLYC_Fp_. The expression altered the HPLC profile from strain ASTA** (Figure S2). As standards are not available for glycosylated carotenoids, we opted for qualitative and quantitative analysis of (glycosylated) carotenoids via mass spectrometry. The monoisotopic mass (m/z) of investigated carotenoids by mass spectrometry are given in Table S2.

The carotenoid production profiles of strains ASTAGLYC_Pa_ and ASTAGLYC_Fp_ are listed in Table 2. In summary both free and glycosylated carotenoids were accumulating under *crtX* expression (Figure S4, Table 2). The main glycosylated carotenoid in both strains was adonixanthin-β-D-diglucoside with a titer of 27 mg L^−1^. ASTAGLYC_Pa_ produced 99.6 mg L^−1^ (10 mg (g CDW)^−1^) of total carotenoids with 40% of glycosylated carotenoids with a content of 39 mg L^−1^ (4.3 mg (g CDW)^−1^). In comparison strain ASTAGLYC_Fp_ produced 88.7 mg L^−1^ (10 mg (g CDW)^−1^) of total carotenoids from which 41% were glycosylated carotenoids (Table 2). Both strains accumulated comparable amounts of glycosylated carotenoids, although with CrtX from *F.* *pelagi* higher production of astaxanthin-β-D-diglucoside was reached with 1.8 mg L^−1^. As the chosen mass spectrometry is not suitable for determination of β-carotene, this carotenoid was determined by HPLC. The strain ASTAGLYC_Pa_ produced 2.2 ± 0.3 mg (g CDW)^−1^ β-carotene, whereas strain ASTAGLYC_Fp_ produced 2.1 ± 0.3 mg (g CDW)^−1^ β-carotene.

**Table 2:** Overview of titer and cellular content of nonglycoslyted, glycosylated (italics) and diglycosylated (bold) carotenoids in strains ASTAGLYC_Pa_ and ASTAGLYC_Fp_.

	ASTAGLYC_Pa_		ASTAGLYC_Fp_	
	Titer [mg L^−1^]	Content [mg (g CDW)^−1^]	Titer [mg L^−1^]	Content [mg (g CDW)^−1^]
β-Carotene*	24 ± 2.6	2.2 ± 0.3	19 ± 1.8	2.1 ± 0.3
Canthaxanthin	14.3 ± 1.9	1.1 ± 0.1	15.5 ± 0.1	1.5 ± 0.2
Adonirubin	3.1 ± 0.1	0.3 ± 0.01	2.8 ± 0.4	0.3 ± 0.04
Adonixanthin	15.7 ± 1.4	1.7 ± 0.1	11.8 ± 0.6	1.3 ± 0.1
Astaxanthin	3.5 ± 0.1	0.4 ± 0.02	3.1 ± 0.3	0.3 ± 0.02
Adonirubin-β-d-glucoside	***0.9*** ± ***0.03***	***0.1*** ± ***0.01***	***0.7*** ± ***0.2***	***0.1*** ± ***0.01***
Adonixanthin-β-d-glucoside	***6.5*** ± ***0.2***	***0.7*** ± ***0.1***	***3.7*** ± ***0.1***	***0.3*** ± ***0.1***
Astaxanthin-β-d-glucoside	***0.4*** ± ***0.03***	***0.4*** ± ***0.01***	***0.5*** ± ***0.06***	***0.05*** ± ***0.01***
Zeaxanthin-β-d-diglucoside	**3.1 ± 0.9**	**0.3 ± 0.1**	**2.8 ± 0.5**	**0.3 ± 0.1**
Adonixanthin-β-d-diglucoside	**27 ± 3.2**	**3.1 ± 0.4**	**27 ± 2.5**	**3.9 ± 0.4**
Astaxanthin-β-d-diglucoside	**1.1 ± 0.01**	**0.1 ± 0.01**	**1.8 ± 0.04**	**0.2 ± 0.01**

β-carotene production (*) is calculated from HPLC measurement (Figure S1), whereas the other carotenoids are measured with mass spectrometry. Cells were grown in 40 g L^−1^ glucose CGXII_opt_ minimal medium for 48 h in a volume of 10 mL in shaking flasks for astaxanthin production. Production yields mean values and standard deviations of three triplicate cultivations are given.

monoglycosylated: boldItalic.

diglycosylated: bold.

## Discussion

In this study the astaxanthin production from *C. glutamicum* was improved by three pathway engineering strategies namely (1) improved precursor supply, (2) improved terminal biosynthesis by an improved fusion-enzyme CrtZ~m~W and (3) a balanced expression of the terminal genes *crtZ* and *crtW*. This enhanced the astaxanthin production fivefold to 3.5 mg g^−1^ CDW (39 mg L^–1^) in shaking flask with astaxanthin as the dominant carotenoid (72%). Cultivation of this ASTA** strain under fed-batch conditions yielded a high cell density with a total carotenoid titer of 421 mg L^−1^ with 103 mg L^–1^ of astaxanthin. In addition, astaxanthin-β-d-diglucoside was produced for the first time in *C. glutamicum* together with other glycosylated C40 carotenoids.

The biosynthesis of the carotenogenic pyrophosphate precursor GGPP (C20) based on IPP and DMAPP is an important step to push the flux from the MEP pathway towards C40 carotenoid biosynthesis. Therefore, the major GGPP synthase IdsA^50^ as well as the isoprenoid pyrophosphate isomerase Idi represent promising overexpression targets that could push the carbon flux towards carotenogenesis and balance the availability of DMAPP and IPP for an improved GGPP biosynthesis. An overexpression of *idi*, *idsA* or *crtBI* as well as the combination of these genes demonstrated to be beneficial for an enhanced astaxanthin production^22^. In this study, it could be shown that both plasmid-based or chromosome-based overexpression of an artificial operon compromising endogenous *idsA* and *idi* from the strong and synthetic P_syn_ promoter resulted in the highest β-carotene content of up to 18 mg (g CDW)^−1^ to date for *C.* *glutamicum*.

From the carotenoid production profile of ASTA* we can deduce that astaxanthin biosynthesis in *C.* *glutamicum* relies on sequential actions of CrtW and CrtZ^57^ that are present in the membrane-fusion enzyme CrtZ ~ W. Balancing of the expression of *crtW* and *crtZ* increased astaxanthin production in other studies^57^. With a second plasmid expressing the β-carotene hydroxylase gene *crtZ* in strain ASTA** the precursor conversion to astaxanthin could be improved to the highest content of 3.5 mg (g CDW)^−1^ in *C.* *glutamicum*, with astaxanthin as dominant carotenoid with 72%, and approximately 22% of β-carotene (Fig. 4). In contrast, the expression of *crtW* could not be identified as the rate-limiting step of astaxanthin biosynthesis as published for other organisms e.g. *Saccharomyces cerevisiae* or *E. coli*^19,58^. This suggests the need for a specific terminal pathway balance between CrtZ and CrtW in the individual host, since the substrate and product specificity of the used enzymes as well as their activities differ. In this study, expression of *crtZ* was identified as the major bottleneck when terminal astaxanthin biosynthetic genes were taken from *F.* *pelagi*.

The intricate relationship between the accumulation of astaxanthin biosynthesis intermediates and the reduced overall carotenoid production supports the hypothesis of a feedback regulation on the upstream biosynthesis pathway. In particular, the difference in the β-carotene production capacity with 18 mg (g CDW)^−1^ in BETA6 as compared to the substantial reduction in total carotenoid contents of 5.8 mg (g CDW)^−1^ in ASTA** (Fig. 4) supports the hypothesis of a feedback inhibition.

In a recent study, alternative lycopene β-cyclases were identified that show a mitigated effect towards a possible feedback inhibition by various oxy-functionalized cyclic C40 carotenoids from astaxanthin biosynthesis, resulting in an improved total carotenoid biosynthesis in *C. glutamicum*^45^. Another study with *H. pluvialis* suggested that free astaxanthin could potentially inhibit its own biosynthesis and therefore the production of astaxanthin variants such as esterified astaxanthin was recommended^56^.

As terminal astaxanthin biosynthesis from β-carotene is complex with 7 possible intermediates that could accumulate (Fig. 1), we identified an improved fusion-enzyme CrtZ ~ W that facilitated the conversion from β-carotene to the final product by 30% (Fig. 3). While flexible linkers could be successfully used for a fusion enzyme comprising CrtZ and CrtW^22^, this study identified a larger linker (20 aa vs. 10 aa) as beneficial for astaxanthin biosynthesis (Fig. 3). The detailed structural mechanisms could be investigated with NMR or X-ray studies in order to rationalize the different structural variances between different linker sizes^59^. Other studies investigated similar strategies for an improved astaxanthin production with the application of a colocalization of CrtZ and CrtW by a RIDD/RIAD complexing^60^. Assembling of enzymes by RIAD-RIDD interaction was superior to traditional linker-based methods^61^. The application of directed evolution and enzyme engineering further enables the optimization of specific enzymes within the biosynthetic pathway for enhanced productivity^21^.

In the broader context, the biotechnological production of astaxanthin offers distinct advantages over traditional petrochemical synthesis methods. In addition, petrochemical processes are far more cost effective, making microbial processes a sustainable and environmentally friendly alternative. The fed-batch fermentation resulted in 103 mg L^−1^ of astaxanthin and 400 mg L^−1^ of total carotenoids with the optimized strain ASTA**, which notably surpassed the astaxanthin production from the non-optimized ASTA* strain in stirred bioreactors (maximum titer of 64 mg L^−1^ and maximum vol. productivity of 0.85 mg L^−1^ h^−1^ achieved by ASTA*)^27^. Here a superior volumetric productivity of 1.5 mg L^−1^ h^−1^ and a yield of 0.34 mg g^−1^ glucose was reached with ASTA**. However, it could be stated that the full potential of the ASTA** strain was not realized in the bioprocess presented here since the astaxanthin content did not reach the 3.5 mg (g CDW)^−1^ of shake flask cultivation. The total amount of astaxanthin compared to the total carotenoids should further be increased, as in the fermentation only 26% of the carotenoids were astaxanthin (Fig. 5), while in the flask cultivation 71% astaxanthin were reached (Fig. 4). Interestingly the precursor profile between shake flask cultivation and fermentation is substantially different. Expression of *crtZ* was identified as the bottleneck in shaked flasks, whereas in the stirred bioreactor the carotenoid profiles of BETA6 (pSH1-*crtZ* ~ m ~ *W*) derivatives looked almost identical (Fig. 4). Although the astaxanthin titer is lower than compared to other organisms, the advantage of the production with *C. glutamicum* is the shorter process time, which increases volumetric productivity in particular in comparison to yeast and algae. The volumetric productivity of astaxanthin production with *C. glutamicum* ASTA** was 1.5 mg L^−1^ h^−1^ (Table 1), whereas the volumetric productivity of yeast *X. dendrorhous* is 0.027 mg L^−1^ h^−1^^62^ or 0.19 mg L^−1^ h^−1^ with algae *H. pluvialis* as production can take up to 12 days^63^. Moreover, recently a downstream processing paper describes the generation of an astaxanthin-containing oleoresin from *C. glutamicum*^64^ and thus complements the microbial strain development.

*C. glutamicum* naturally accumulates the cyclic C50 carotenoid decaprenoxanthin primarily as a di-glucoside due to the activity of an endogenous glycosyltransferase which can glycosylate cyclic C50 but not C40 carotenoids, like zeaxanthin^44^. Producing glycosylated carotenoids using microbial hosts in biotechnology offers a scalable, controlled, and sustainable approach to generate these modified compounds. Glycosylated astaxanthin was shown for the first time in *E. coli*^34^. In another study in *E. coli* seven C40 carotenoid glucosides including astaxanthin di-glucoside were produced with a total yield of 8.1 mg/L, determined by mass spectrometry^37^. In this study, we established production of glycosylated C40 carotenoids through expression of *crtX* from *P.* *ananatis* or *crtX* from *F.* *pelagi* (uniprot: A0A0P0Z8H5). To the best of our knowledge CrtX from *F.* *pelagi* was used for the first time to produce glycosylated carotenoids in a heterologous bacterial host resulting in a 47% conversion to glycosylated intermediates. As HPLC was not suitable for determination of intermediates from the complex biosynthesis of glycosylated C40 carotenoid (Figure S1), mass spectrometry was chosen for analysis of ASTAGLYC_Pa_ and ASTAGLYC_Fp_ (Table 1, Figure S3 and S4) and revealed that the intermediate profile became even more complex compared with the ASTA** strain. In *Yarrowia lipolytica* this challenge of complexity was tackled by the establishment of an alternative plant-derived astaxanthin biosynthesis from *Adonis aestivalis*^20^ resulting in astaxanthin as the exclusive hydroxylated carotenoid due to the sequential activity of carotenoid 4-hydroxy-β-ring 4-dehydrogenase (HBFD) and carotenoid β-ring 4-dehydrogenase (CBFD). It was recently demonstrated that also the UDP-glucose pool can be enhanced by overexpression of the endogenous UDP-d-glucose pathway genes, phosphoglucomutase (*pgm*) and UDP-d-glucose pyrophosphorylase (*galU1*) together with the heterologous expression of *ydhE* from *Bacillus licheniformis*^65^ and this strategy might also improve the biosynthesis of glycosylated carotenoids.

## Methods

### Bacterial strains, media and growth conditions

Strains and plasmids used in this study are listed in Table S3. Chemicals were delivered by Carl Roth (Karlsruhe, Germany) if not stated differently. *E. coli* DH5α cells were used for cloning and were cultivated at 37 °C in LB medium^66^. Precultures of *C. glutamicum* strains were grown in LB medium supplemented with 10 g L^−1^ glucose overnight and addition of antibiotics. The main cultures of *C. glutamicum* for the screening of carotenoid production were grown in CGXII minimal medium^67^ or in CGXII minimal media with optimized trace salts concentrations (25 g L^−1^ FeSO_4_ × 7H_2_O, 2.5 g L^−1^ MnSO_4_ x H_2_O, 1.18 g L^−1^ ZnSO_4_ × 7H_2_O, 0.15 g L^−1^ CuSO_4_ × 5H_2_O, 0.015 g L^−1^ NiCl_2_ × 6H_2_O)^52^, called CGXII_opt_, both supplemented with 40 g L^−1^ glucose and supplemented with 1 mM IPTG for induction if needed after washing in the minimal medium. Cultures were inoculated to an initial OD_600nm_ of 1 using a Shimadzu UV-1202 spectrophotometer (Duisburg, Germany). Cultivations of *C. glutamicum* were performed at 30 °C in a volume of 10 mL in 100 mL flasks with two baffles shaking at 120 rpm on a rotary shaker. Tetracycline, Kanamycin and Spectinomycin (VWR, Darmstadt, Germany) were added if appropriate to respective concentrations of 5 μg mL^−1^, 25 μg mL^−1^ and 100 μg mL^−1^ in *C. glutamicum* cultures and in *E. coli* cultures.

### Cloning of expression plasmids and bacterial transformations

The oligonucleotides used in this study were obtained from Metabion (Planegg/Steinkirchen, Germany) and are listed in Table S4. Expression plasmids were constructed in *E. coli* DH5α. First target genes were amplified with a high-fidelity PCR (All-in HiFi, highQu, Kraichtal, Germany). The PCR amplicon was purified with a PCR and gel extraction kit (Macherey–Nagel, Düren, Germany). The gene fragments were cloned into with BamHI (NEB, Frankfurt, Germany) digested and dephosphorylated (Antarctic phosphatase, New England Biolabs, Frankfurt, Germany) expression vectors by Gibson-Assembly. The concentration of DNA was measured with a Spectrophotometer ND-1000 (Thermo Fisher Scientific, Schwerte, Germany). *E. coli* DH5α cells were transformed by heat shock after preparation of CaCl_2_ competent cells^67^. Transformants were screened by colony-PCR and plasmids were isolated by plasmid miniprep kit (GeneJET, Thermo Fisher Scientific, Schwerte, Germany). Constructed vectors were confirmed by sequencing with oligonucleotides from Table S4. *C. glutamicum* cells were transformed by electroporation^68^. Standard genetic procedures were performed as described previously^69^. All constructed plasmids included an individual optimized RBS for each expressed gene. RBS design was aided by RBS Calculator (version 2.0, https://salislab.net/software/).

### Construction of genomic replacement mutants of *C. glutamicum*

For deletion of the *idi* gene with promoter region and insertion of the promotor P_syn_ together with *idi* and *idsA* in a synthetic operon, the suicide vector pK19*mobsacB* was used (Table S3). The promotor region of *idi* was identified from publication Pfeifer-Sancar 2013^55^. The genomic flanking regions of *idi* were amplified from the genomic DNA of *C. glutamicum* WT using the oligonucleotide listed in Table S4 together with the insertion fragments of *idi* and *idsA* together with an optimized RBS. The PCR amplicons were purified, linked by crossover PCR, and subsequently cloned into a *BamHI*-restricted pK19*mobsacB*. Deletion and insertion were achieved by two-step homologous recombination using the respective deletion vector, as previously described^67^. Integration of the vector into one of the gene-flanking regions represents the first recombination event and was selected via kanamycin resistance. Integration of the vector into the genome results in sucrose sensitivity due to levansucrase, encoded by *sacB*. Selection for the second recombination event, loss of the vector, was carried out via sucrose resistance. Deletion and insertion was verified via sequencing with primers V479 and V480 (Table S4).

### Fed-batch fermentation of carotenoid producers

Fermentation of *C. glutamicum* strains was performed in a fed-batch cultivation process in a bioreactor with a total volume of 3.7 L (KLF, Bioengineering AG, Switzerland). The stirrer to reactor diameter ratio was 0.39 and the aspect ratio of the reactor was 2.6:1.0. Three six-bladed Rushton turbines were placed on the stirrer axis with a distance of 6, 12 cm and 18 cm from the bottom of the reactor. For inoculation of the bioreactor a first pre-culture was grown in 50 mL LB medium with addition of 10 g L^−1^ glucose and 25 µg mL^−1^ kanamycin and 5 μg mL^−1^ tetracycline in a 500 mL shake flask. A second pre-culture in 200 mL CGXII minimal medium, supplemented with 40 g L^−1^ glucose with 25 µg mL^−1^ kanamycin and 5 μg mL^−1^ tetracycline in 2 L shake flasks was inoculated with 1% (v/v) of the first preculture. The precultures were grown overnight at 30 °C and 120 rpm.

The fed-batch fermentations were performed with a head space overpressure of 0.5 bar. The temperature was kept at 30 °C during the fermentations. A pH of 8.0 was automatically maintained by the addition of 10% (v/v) H_3_PO_4_ and 25% (v/v) NH_3_. The fed-batch fermentations were performed with an initial working volume of 1 L HCDC medium^70^, inoculated to an OD_600 nm_ of 1 with the second pre-culture. 1 L 600 g L^−1^ glucose was used as feed medium. A steady airflow of 0.25 to 3.5 NL min^−1^ was maintained from the bottom through a ring sparger, the airflow was increased manually during the process when oxygen supply became limiting. An automatic control increased the stirrer speed from 400 to 1500 rpm every time the relative dissolved oxygen saturation (rDOS) fell below 30%. The feed pump was primed when the rDOS fell below 60% for the first time. The feed pump activated every time the rDOS exceeded 60% and stopped when it subsequently fell below 60%, to prevent oversaturation with glucose. Foam formation during the fermentation was controlled by addition of 0.6 mL L^−1^ antifoam 204 to the batch medium and controlled addition of 60 ml antifoam 204 via an antifoam probe during the feeding phase.

Sampling during the fermentations was conducted with autosamplers and cooled storage at 4 °C until further use. A Shimadzu UV-1202 spectrophotometer (Duisburg, Germany) was used for OD_600 nm_ measurements.

### Carotenoid extraction and quantification

Carotenoids were extracted as described before^45^. The Agilent 1200 series system (Agilent Technologies, Waldbronn, Germany) was used with a reversed phase precolumn (LiChrospher 100 RP18 EC-5, 40 × 4 mm) (CS-Chromatographie, Langerwehe, Germany) and a reversed phase main column (LiChrospher 100 RP18 EC-5, 125 × 4 mm) (CS-Chromatographie, Langerwehe, Germany) and methanol:water (9:1) (A) and methanol (B) were used as mobile phases. Carotenoids were detected with a diode array detector (DAD) through recording of the UV/visible (Vis) spectrum. The injection volume was 50 µL and a gradient at a flow rate of 1.5 mL min^−1^ was used as the following; 0 min B: 0%, 10 min B: 100%, 32.5 min B: 100%. For quantification the signal of the extract at wavelength λ_max_ 471 nm was used. Standard calibration curves were generated with lycopene (ExtraSynthese, Genay, France), β-carotene (Sigma-Aldrich, St. Louis, USA), canthaxanthin (VWR, Darmstadt, Germany), zeaxanthin (ExtraSynthese, Genay, France), echinenone (Sigma-Aldrich, St. Louis, USA), adonirubin (CaroteNature, Münsingen, Schwitzerland), adonixanthin (CaroteNature, Münsingen, Schwitzerland) and astaxanthin (Sigma-Aldrich, St. Louis, USA) to quantify carotenoid titers. All standards were dissolved in chloroform according to their solubility and diluted in methanol:acetone (7:3) containing 0.05% BHT.

### Orbitrap quantification of glycosylated carotenoids

Carotenoid analysis was performed using a Vanquisch Flex UHPLC system (Thermo Fisher Scientific, Schwerte, Germany) coupled to an Orbitrap Exploris 120 mass spectrometer (Thermo Fisher Scientific, Schwerte, Germany). For this purpose, 5 µl of the carotenoid extract, as described in the chapter before, was injected and separated using a Eurosphere II C18 150 × 2 mm HPLC column (Knauer, Berlin, Germany). The analysis was performed at 40°C oven temperature and a flow rate of 0.4 mL min^−1^. The mobile phase consisted of water (A) and acetonitril (B), each with 0.1% formic acid added. The following gradient profile was used for separation: 0 min 10% B, 20 min 95% B, 21 min 10% B, and 30 min 10% B. The mass spectrometer was operated with the following parameters: 150–1200 m/z scan range, positive polarity, 120,000 resolution, 3400 V capillary voltage, 50 arb sheath gas, 10 arb auxiliary gas, 1 arb sweep gas, 325°C for ion transfer tube and vaporizer. EASY-IC mode was activated in scan-to-scan mode for internal mass calibration.

Identification and peak area determination of carotenoids were performed using Freestyle 1.3 software (Thermo Fisher Scientific, Schwerte, Germany). Extraction ion chromatograms (EIC) of the individual [M + H]^+^ masses of the carotenoids were generated with a variance of 2 ppm for correct identification. When available, the determined retention times were matched with those of the measured standards. EICs were also used to determine the peak areas of the carotenoids, but here with a mass variance of 5 ppm.

Carotenoid concentrations were calculated based on the peak area of each compound extracted by their corresponding m/z. Standard curves were generated for the five chemical standards with extracted-ion chromatogram (EIC) peak areas: astaxanthin (Sigma-Aldrich, St. Louis, USA), adonixanthin (CaroteNature, Münsingen, Schwitzerland), adonirubin (CaroteNature, Münsingen, Schwitzerland), zeaxanthin (Carl Roth GmbH, Karlsruhe, Germany) and canthaxanthin (VWR, Darmstadt, Germany). For those carotenoids without standards, the concentration was calculated based on the relative peak area to its close compartment, under the assumption that the closely related compounds can be measured similarly. For example, astaxanthin glucosides were quantifies as astaxanthin equivalents using an astaxanthin standard.

### Supplementary Information

Supplementary Information.

## Data Availability

The datasets used and/or analysed during the current study available from the corresponding author on reasonable request.
