# Supplementary material for: Enhancing astaxanthin biosynthesis and pathway expansion towards glycosylated C40 carotenoids by Corynebacterium glutamicum
Source: Sci Rep. 2024 Apr 6;14:8081. doi: 10.1038/s41598-024-58700-9 (PMC10998873; doi:10.1038/s41598-024-58700-9)
Supplement: Supplementary file 1 — Supplementary Information. [file 41598_2024_58700_MOESM1_ESM.docx]

**Supplementary materials**

**Enhancing astaxanthin biosynthesis and pathway expansion towards glycosylated C40 carotenoids by *Corynebacterium glutamicum***

Vanessa L. Göttl^1^, Florian Meyer^1^, Ina Schmitt^1^, Marcus Persicke^2,3^, Petra Peters-Wendisch^1^,
Volker F. Wendisch^1^ and Nadja A. Henke^1,4*^

^1^ Genetics of Prokaryotes, Faculty of Biology & CeBiTec, 33615 Bielefeld University, Germany;

^2^ CeBiTec, Bielefeld University, 33615 Bielefeld, Germany;

^3^ Omics Core Facility – Proteom-Metabolom Unit (in development), Bielefeld University, 33615 Bielefeld, Germany;

^4^CZS Junior Research Group, Microsystems in Bioprocess Engineering, Department of Process Engineering in Life Sciences, Karlsruhe Institute of Technology, 76131 Karlsruhe, Germany

*****Correspondence: nadja.henke@kit.edu; Tel.: +49-721 608 45208

**Figure S1: Fed-Batch fermentations of astaxanthin producer strains.** Fed-batch fermentation with C. glutamicum astaxanthin producer strains grown in 1 L HCDC as batch medium, fed with 1 L 600 g L^-1^ glucose at pH 8 with an initial aeration rate of 0.25 vvm. Given for all fermentations are the following fermentation parameters over time: Astaxanthin titers (red squares), OD_600 nm_ (green dots), feed intake (purple line), agitator speed (grey line), moving average rDOS (dark blue line), and aeration rate (light blue line). Secondly, HPLC results of all produced carotenoids in fed-batch fermentations of C. glutamicum strains over time together with produced biomass (black squares). **(A):** Fermentation process and HPLC data of strain BETA6 (pSH1-crtZ~W) (pECXT-Psyn-crtZ-crtW). **(B):** Fermentation process and HPLC data of strain BETA6 (pSH1-crtZ~W) (pECXT-Psyn-crtW). **(C):** Fermentation process and HPLC data of strain BETA6 (pSH1-crtZ~W) (pECXT-Psyn-crtZ) = ASTA**.

**Figure S2: HPLC chromatograms of carotenoids extracted from C. glutamicum strain ASTA**, ASTAGLYC_Pa_ and ASTAGLYC_Fp_.** Shown are HPLC elution profiles for extracts of C. glutamicum strains. **(A)**: Chromatogram of astaxanthin producing ASTA** strain. **(B)**: of strain ASTA** expressing the plasmid pEKEx3-crtX_Pa_ (ASTAGLYC_Pa_). **(C)**: of strain ASTA** expressing the plasmid pEKEx3-crtX_Fp_ (ASTAGLYC_Fp_). For quantification the extracted wavelength chromatogram at λ_max_ 471 nm was used. The numbers in the chromatogram represent the elution profile of the identified carotenoids: 1: astaxanthin, 2: adonirubin, 3: canthaxanthin, 4: echinenone and 5: β-carotene.

**Figure S3: Mass spectra of produced astaxanthin and glycosylated astaxanthin.** Mass spectra of astaxanthin and its glucosides (left) and extracted ion chromatograms of astaxanthin and its glucosides in strain ASTAGLYC_Pa_ (right). **A:** astaxanthin **B**: astaxanthin-β-d-glucoside and **C:** astaxanthin-β-d-diglucoside.

**Figure S4**: **Distribution of glycosylated C40 carotenoids in *C. glutamicum*.** Genes encoding for the enzymes are depicted next to the reaction. *crtZ*: β-carotene hydroxylase, *crtW*: β-carotene ketolase and *crtX*: glycosyltransferase. Depicted in blue are the reactions from β-carotene ketolase. Depicted in green are the reactions from β-carotene hydroxylase. Depicted in red are the reactions from glycosyltransferase. Production contents of glycosylated carotenoids measured with mass spectrometry are given for ASTAGLYC_Pa_ in red and ASTAGLYC_Fp_ in red in brackets. Thicker arrows indicate higher production of the resulting product. Cells were grown in 40 g L^-1^ glucose CGXII_opt_ minimal medium for 48 h in a volume of 10 mL in shaking flasks for astaxanthin production.

**Table S1:** **Increased β-carotene production by expression of idi and idsA and transfer to genomic integration with strain BETA6.** β-carotene production and max. OD_600_ of engineered C. glutamicum strains. The β-carotene production of strain BETA4 was compared to a strain expressing additional idi and idsA on plasmid pECXT-P_syn_ and strain BETA6. Production contents, max. OD_600_, mean values and standard deviations of three triplicate cultivations are given. Cells were grown in 40 g L^-1^ glucose CGXII_opt_ minimal medium for 48 h in a volume of 10 mL in shaking flasks.

| *C. glutamicum* strains | max. OD_600_ | Titer  [mg L^-1^] | β-carotene production  [mg (g CDW)^-1^] |
| --- | --- | --- | --- |
| BETA4 | 53.3 ± 0.9 | 188 | 14.1 ± 0.6 |
| BETA4 (pECXT-P_syn_-*idi-idsA*) | 52.3 ± 1.8 | 259 | 19.8 ± 2.0 |
| BETA6 | 45.0 ± 1.3 | 204 | 18.0 ± 2.0 |

**Table S2.** Chemical formula and monoisotopic mass of non-glycosylated and glycosylated carotenoid which were investigated with mass spectrometry.

| Compound | Chemical formula | monoisotopic mass (m/z) |
| --- | --- | --- |
| **β-carotene** | C_40_H_56_ | 536.438 |
| **Echinenone** | C_40_H_54_O | 550.417 |
| **β-cryptoxanthin** | C_40_H_56_O | 552.433 |
| **Canthaxanthin** | C_40_H_52_O_2_ | 564.397 |
| **Hydroxyechinenone** | C_40_H_54_O_2_ | 566.412 |
| **Zeaxanthin** | C_40_H_56_O_2_ | 568.428 |
| **Adonirubin** | C_40_H_52_O_3_ | 580.392 |
| **Adonixanthin** | C_40_H_54_O_3_ | 582.407 |
| **Astaxanthin** | C_40_H_54_O_4_ | 596.387 |
| **β-cryptoxanthin-β-D-glucoside** | C_46_H_66_O_6_ | 714.486 |
| **Hydroxyechinenone-β-D-glucoside** | C_46_H_64_O_7_ | 728.465 |
| **Zeaxanthin-β-D-glucoside** | C_46_H_66_O_7_ | 730.481 |
| **Adonirubin-β-D-glucoside** | C_46_H_62_O_8_ | 742.444 |
| **Adonixanthin-β-D-glucoside** | C_46_H_64_O_8_ | 744.460 |
| **Astaxanthin-β-D-glucoside** | C_46_H_62_O_9_ | 758.439 |
| **Zeaxanthin-β-D-diglucoside** | C_52_H_76_O_12_ | 892.534 |
| **Adonixanthin-β-D-diglucoside** | C_52_H_74_O_13_ | 906.513 |
| **Astaxanthin-β-D-diglucoside** | C_52_H_72_O_14_ | 920.492 |

**Table S3.** Strains and plasmids used in this study.

| **Strain** | **Characteristics** | **Reference** |
| --- | --- | --- |
|  | ***C. glutamicum* strains** |  |
| BETA4 | MB001 derivative with deletion of *crtYEb* (cg0717-0719) and *crtR* (cg0725) and integration of P*tuf-dxs*, P*tuf-crtEBI*, P*tuf* -*crtY_Pa_* | [1] |
| BETA6 | BETA4 derivative with genomic integration of P_syn_-*idsA-idi* in the *idi* locus (cg2531) | this work |
| ASTA* | BETA4 derivative with pSH1-*crtZ~W_Fp_* | [2] |
| ASTA** | BETA6 derivative with pSH1-*crtZ~~W_Fp_* with middle linker size and pECXT-Psyn-*crtZ_Fp_* | this work |
| BETA6 (pSH1-*crtZ~W*) (pECXT-P_syn_-*crtW*) | BETA6 derivative with pSH1-*crtZ~~W_Fp_* with middle linker size and pECXT-Psyn-*crtW_Fp_* | this work |
| BETA6 (pSH1-*crtZ~W*) (pECXT-P_syn_-*crtZ*-*crtW*) | BETA6 derivative with pSH1-*crtZ~~W_Fp_* with middle linker size and pECXT-Psyn-*crtZ_Fp_-crtW_Fp_* | this work |
| ASTAGLYC_Pa_ | ASTA** derivative with pEKEx3-*crtX_Pa_* | this work |
| ASTAGLYC_Fp_ | ASTA** derivative with pEKEx3-*crtX_Fp_* | this work |
|  | **other strains** |  |
| *E. coli* DH5$\alpha$ | F^-^thi^-1^ *endA*1 *hsdr*17(r-, m-) *supE*44 Δ*lacU*169 (Φ80*lacZ*ΔM15) *recA*1 *gyrA*96 | [3] |
|  | **Plasmids** |  |
| pECXT-P_syn_ | pECXT99A derivative for constitutive expression from synthetic P*syn* promoter | [4] |
| pSH1 | Km^R^, P*_tu_*f, pHM519 *oriV_Cg_*, *C. glutamicum/E. coli* expression shuttle vector | [1] |
| pEKEx3 | SpecR, *PtrclacIq*, pBL1 *oriV_Cg_*, *C. glutamicum*/*E. coli* expression shuttle vector | [5] |
| pK19*mobsacB* | KmR; *E. coli*/*C. glutamicum* shuttle vector for construction of insertion and deletion mutants in *C. glutamicum* (pK18 *oriVEc sacB lacZα*) | [6] |
| pSH1-*crtZ~W* | pSH1 derivative for constitutive expression of *crtZ~W* encoding for a fusion protein comprising CrtZ and CrtW from *F. pelagi* with a linker size of 10 aa | [2] |
| pSH1-*crtZ~~W* | pSH1 derivative for constitutive expression of *crtZ~W* encoding for a fusion protein comprising CrtZ and CrtW from *F. pelagi* with a middle linker size (20 aa) | this work |
| pSH1-*crtZ~~~W* | pSH1 derivative for constitutive expression of *crtZ~W* encoding for a fusion protein comprising CrtZ and CrtW from *F. pelagi* with a large linker size (29 aa) | this work |
| pECXT-Psyn-*idi-idsA* | pECXT-P_syn_ derivative for constitutive expression of *idi* (cg2531) and *idsA* (cg2384) | this work |
| pECXT-Psyn-*crtZ*_Fp_ | pECXT-P_syn_ derivative for constitutive expression of *crtZ* from *F. pelagi* | this work |
| pECXT-Psyn-*crtW*_Fp_ | pECXT-P_syn_ derivative for constitutive expression of *crtW* from *F. pelagi* | this work |
| pECXT-Psyn-*crtZ*_Fp_-*crtW*_Fp_ | pECXT-P_syn_ derivative for constitutive expression of *crtZ* and *crtW* from *F. pelagi* in a synthetic operon | this work |
| pK19*mobsacB*Δ*idi*::P_syn_-*idsA-idi* | pK19*mob*s*acB* derivative for the deletion of the *idi* locus together with the promotor region and integration of *idsA* and *idi* in a synthetic operon with P_syn_ promotor | this work |
| pEKEx3-*crtX*_Fp_ | pEKEx3 derivative for constitutive expression of *crtX* from *F. pelagi* | this work |
| pEKEx3-*crtX*_Pa_ | pEKEx3 derivative for constitutive expression of *crtX* from *P. ananatis* | this work |

**Table S4.** Oligonucleotides used in this study.

| Oligonucleotide | Sequence (5’🡪 3’) | Used for |
| --- | --- | --- |
| V163 | GCGCCTGATGCGGTATTTTC | forward sequencing primer for pECXT-P_syn_ |
| 1135 | TACTGCCGCCAGGCAAATTC | reverse sequencing primer for pECXT-P_syn_ and pSH1 |
| 1129 | ACCGGCTCCAGATTTATCAG | forward sequencing primer for pSH1 |
| 196 | CGCCAGGGTTTTCCCAGTCACGAC | forward sequencing primer for pK19mobsacB |
| 197 | AGCGGATAACAATTTCACACAGGA | reverse sequencing primer for pK19mobsacB |
| 1127 | GCGCCGACATCATAACGG | forward sequencing primer for pEKEx3 |
| 1128 | GGCGTTTCACTTCTGAGTTCGG | reverse sequencing primer for pEKEx3 |
| V172 | CAAGCGCCGCGCGATAATTAAAAGGTTGTATTTTTATGACGATCTGGACTCTCTACTACGTC | forward primer for amplification of *crtZ* and RBS |
| V173 | CGAGCTCGGTACCCGGGCAAGCGCCGCGCG | forward primer for amplification of *crtZ* and pECXT-Psyn |
| V174 | CAGGTCGACTCTAGAGGATCTTACCGAACCGGCGCG | reverse primer for amplification of *crtZ* and pECXT-Psyn |
| V554 | CGCTCTTAAGAGCGCGTATTAATTCGAACAAAGGAGGATTTTTATGACCCTCAGCCCAACC | forward primer for amplification of *crtW* and RBS |
| V555 | GGTTCCATGGAATTCGAGCTCGGTACCCGGGTACGCTCTTAAGAGCGCGTATTAATTCG | forward primer for amplification of *crtW* and pECXT-Psyn |
| V556 | GCCAAGCTTGCATGCCTGCAGGTCGACTCTAGAGGATCTTAGGACTGGCGAGTATGCGG | reverse primer for amplification of *crtW* and pECXT-Psyn |
| V205 | AAGGTCTTGAAAGATCTTACATCAAAAACCAAAAGGAGTTGTTTTATGACGATCTGGACTCTCTACTACG | forward primer for amplification of *crtZ* and RBS |
| V206 | GCATGCCTGCAGGTCGACTCTAGAGTGCAAAGGTCTTGAAAGATCTTACATCAAAAACC | forward primer for amplification of *crtZ* and pSH1 |
| V208 | ACCTGGACCGCCGGAACCGCCACCGCCGGAACCGCCACCGCCGGAACCGCCACCGCCCCGAACCGGCGCG | reverse primer for amplification of *crtZ* with middle linker |
| V209 | GGTTCCGGCGGTGGCGGTTCCGGCGGTGGCGGTTCCGGCGGTCCAGGTTCCACCCTCAGCCCAACCTCAC | forward primer for amplification of *crtW* with middle linker |
| V314 | CGACGGCCAGTGAATTCGAGCTCGGTACCCGGGGATCTTAGGACTGGCGAGTATGCGG | reverse primer for amplification of *crtW* and pSH1 |
| V296 | GCCGGAACCGCCGGAACCGCCACCGCCGGAACCCCGAACCGGCGCGTG | reverse primer for amplification of *crtZ* with large linker |
| V211 | GGTTCCGGCGGTGGCGGTTCCGGCGGTGGCGGTTCCGGCGGTGGCGGTTCCACCCTCAGCCCAACCTCAC | forward primer for amplification of *crtW* with large linker |
| V297 | CCGCCACCGCCGGAACCGCCACCGCCGGAACCGCCACCGCCGGAACCGCCACCGCCGGAACCGCCGG | reverse primer for amplification of *crtZ* with large linker |
| V345 | CGGATATAGCAACCCGAACATAGGGGTAAAAAGGAGGAATTTGATGTCTAAGCTTAGGGGCATGACTAC | forward primer for amplification of *idi* with and pECXT-P_syn_ |
| V347 | GTTGTTAGGGAACGCGGGCCCCTGGGCCAGCCTTGCAATTCGCGCGTTACTCTGCGTCAAACGCTTCCAG | reverse primer for amplification of *idi* |
| V349 | GGGGCCCGCGTTCCCTAACAACAACAAAAGGATTATTTTTATGAAGGACGTCTCCTTGAGCAG | forward primer for amplification of *idsA* |
| V350 | GCCAAGCTTGCATGCCTGCAGGTCGACTCTAGAGGATCCTACATCCGACGTTCGGTTGAGC | forward primer for amplification of *idsA* and pECXT-P_syn_ |
| V472 | CGCCAAGCTTGCATGCCTGCAGGTCGACTCTAGAGAAGGCGATTTTGGATCCGGTG | amplification of flanking region *idi* |
| V473 | GAGCTCGAATTCCATGGAACCATTATAACACAGATTCAAATTAATGTCAATTAGCCCCTCACCCAGTCAC | amplification of flanking region *idi* |
| V474 | GGACTCGCCGAAGAAAGGAGGTTTCAATTGAAGGACGTCTCCTTGAGCAG | amplification of *idsA* |
| V475 | GAATCTGTGTTATAATGGTTCCATGGAATTCGAGCTCGGTACCCGGGTGGACTCGCCGAAGAAAGGAG | amplification of *idsA* |
| V476 | GGGCCTCCTTTCGTACCCGGGGATCCCTACATCCGACGTTCGGTTGAG | amplification of *idsA* |
| V477 | GGATCCCCGGGTACGAAAGGAGGCCCTTCAATGACTACTGAGGTTGAACTGGTTG | amplification of *idi* and flanking region of *idi* |
| V478 | CGGCCAGTGAATTCGAGCTCGGTACCCGGGGATCGGAAGGCGAAGCCCACATC | amplification of *idi* and flanking region of *idi* |
| V479 | CCTTCGTTGTCGTTGGATGCG | verification of the  *idi* deletion and insertion |
| V480 | CTCCGGGGCTAACTACACC | verification of the  *idi* deletion and insertion |
| HA55 | CCGTGGAGAAAGCCAGATTTGAAAGGAGGCCCTTCAGATGAGCCATTTCGCGGCG | forward primer for amplification of *crtX*_Pa_ and pEKEx3 |
| HA56 | GATGTTAGGAATCTAAAAAAGAAAAGTTATAATGCGGTTGCATAGCCG | reverse primer for amplification of *crtX*_Pa_ and pEKEx3 |
| HA57 | CCGTGGAGAAAGCCAGATTTGAAAGGAGGCCCTTCAGATGGCACGCATAGCTTTGGT | forward primer for amplification of *crtX_Fp_* and pEKEx3 |
| HA58 | GATGTTAGGAATCTAAAAAAGAAAAGTTATGACCTCGCAAATAAGCCGA | reverse primer for amplification of *crtX_Fp_* and pEKEx3 |

**References:**

1. Henke, N.A.; Heider, S.; Peters-Wendisch, P.; Wendisch, V. Production of the Marine Carotenoid Astaxanthin by Metabolically Engineered *Corynebacterium Glutamicum*. *Marine Drugs* **2016**, *14*, 124, doi:10.3390/md14070124.

2. Henke, N.A.; Wendisch, V.F. Improved Astaxanthin Production with *Corynebacterium Glutamicum* by Application of a Membrane Fusion Protein. *Mar. Drugs* **2019**, *17*, 1–12, doi:10.3390/md17110621.

3. Hanahan, D. Studies on Transformation of *Escherichia Coli* with Plasmids. *J. Mol. Biol.* **1983**, *166*, 557–580, doi:10.1016/S0022-2836(83)80284-8.

4. Henke, N.A.; Krahn, I.; Wendisch, V.F. Improved Plasmid-Based Inducible and Constitutive Gene Expression in *Corynebacterium Glutamicum*. *Microorganisms* **2021**, *9*, 204, doi:10.3390/microorganisms9010204.

5. Stansen, C.; Uy, D.; Delaunay, S.; Eggeling, L.; Goergen, J.-L.; Wendisch, V.F. Characterization of a *Corynebacterium Glutamicum* Lactate Utilization Operon Induced during Temperature-Triggered Glutamate Production. *Appl Environ Microbiol* **2005**, *71*, 5920–5928, doi:10.1128/AEM.71.10.5920-5928.2005.

6. Schäfer, A.; Tauch, A.; Jäger, W.; Kalinowski, J.; Thierbachb, G.; Pühler, A. Small Mobilizable Multi-Purpose Cloning Vectors Derived from the *Escherichia Coli* Plasmids pK18 and pK19: Selection of Defined Deletions in the Chromosome of *Corynebacterium Glutamicum*. *Gene* **1994**, *145*, 69–73, doi:10.1016/0378-1119(94)90324-7.
